# Supplementary material for: Biologically indeterminate yet ordered promiscuous gene expression in single medullary thymic epithelial cells
Source: EMBO J. 2019 Oct 28;39(1):e101828. doi: 10.15252/embj.2019101828 (PMC6939203; doi:10.15252/embj.2019101828)
Supplement: Supplementary file 1 — Appendix [file EMBJ-39-e101828-s001.pdf]

Table of Contents

Appendix Figure S1: FACS gating strategy to identify GP2+ and TSPAN8+ mTEC..........2

Appendix Figure S2: Profile of single cell mTEC libraries...........3

Appendix Figure S3. Cell sub-populations are robustly identified across multiple TEC datasets from the literature..........4

Appendix Figure S4. Contribution to mTEC clusters...........5

Appendix Figure S5. Contribution to mTEC clusters...........6

Appendix Figure S6. Expression of selected genes across the dataset..........7

Appendix Figure S7. mTEC expressing the enhanced TRAs Gp2 and Tspan8 have features of terminal differentiation..........8

Appendix Figure S8. Enriched pathways in mTEC clusters...........9

Appendix Figure S9: Spatial analysis of Gp2 distribution shows that Gp2+ mTEC are randomly distributed within the thymic medulla..........10

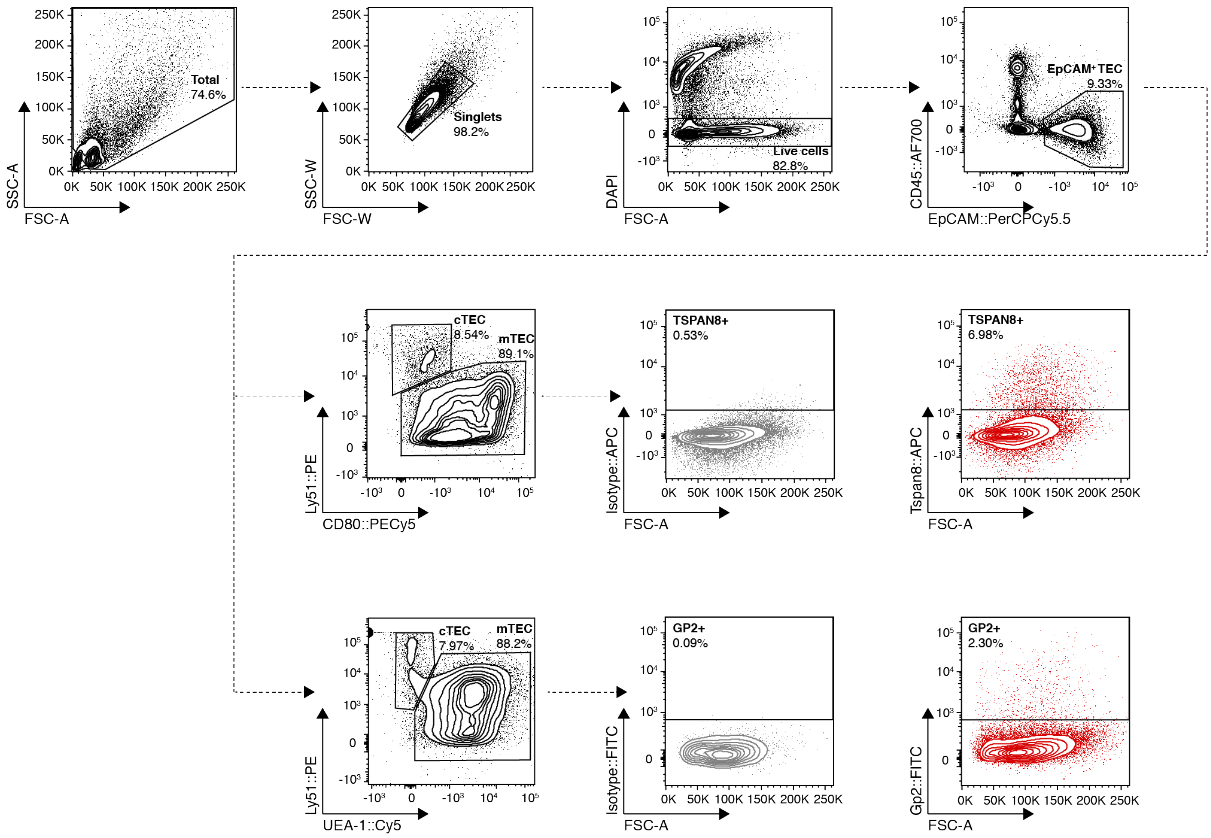

Appendix Figure S1: FACS gating strategy to identify GP2+ and TSPAN8+ mTEC
In order to identify mTEC cells were sequentially gated for forward and side scatter characteristics, singlets, and live cells. TECs were identified within live cells as CD45-EpCAM+ and within this population mTECs were selected as Ly51-UEA1+ or Ly51-. The gates for GP2 and TSPAN8 positivity (red) were set on the basis of an isotope control antibody (grey).

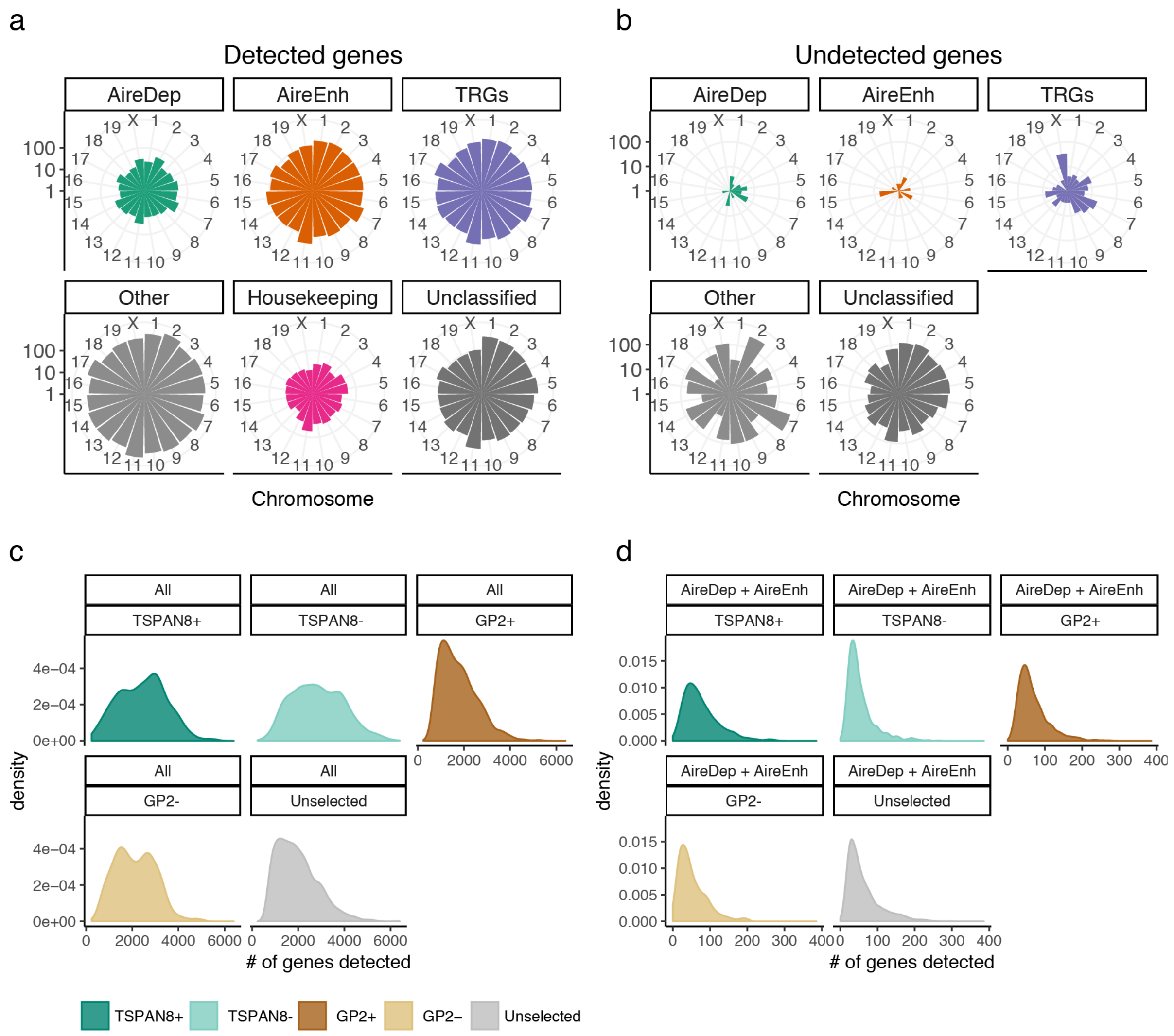

Appendix Figure S2: **Profile of single cell mTEC libraries**.
(a, b) Location of detected (a) and undetected(b) genes across the genome separated by expression category. (c) The number of detected genes per cell separated by experimental condition. (d) The number of detected AIRE-regulated genes per cell separated by experimental condition

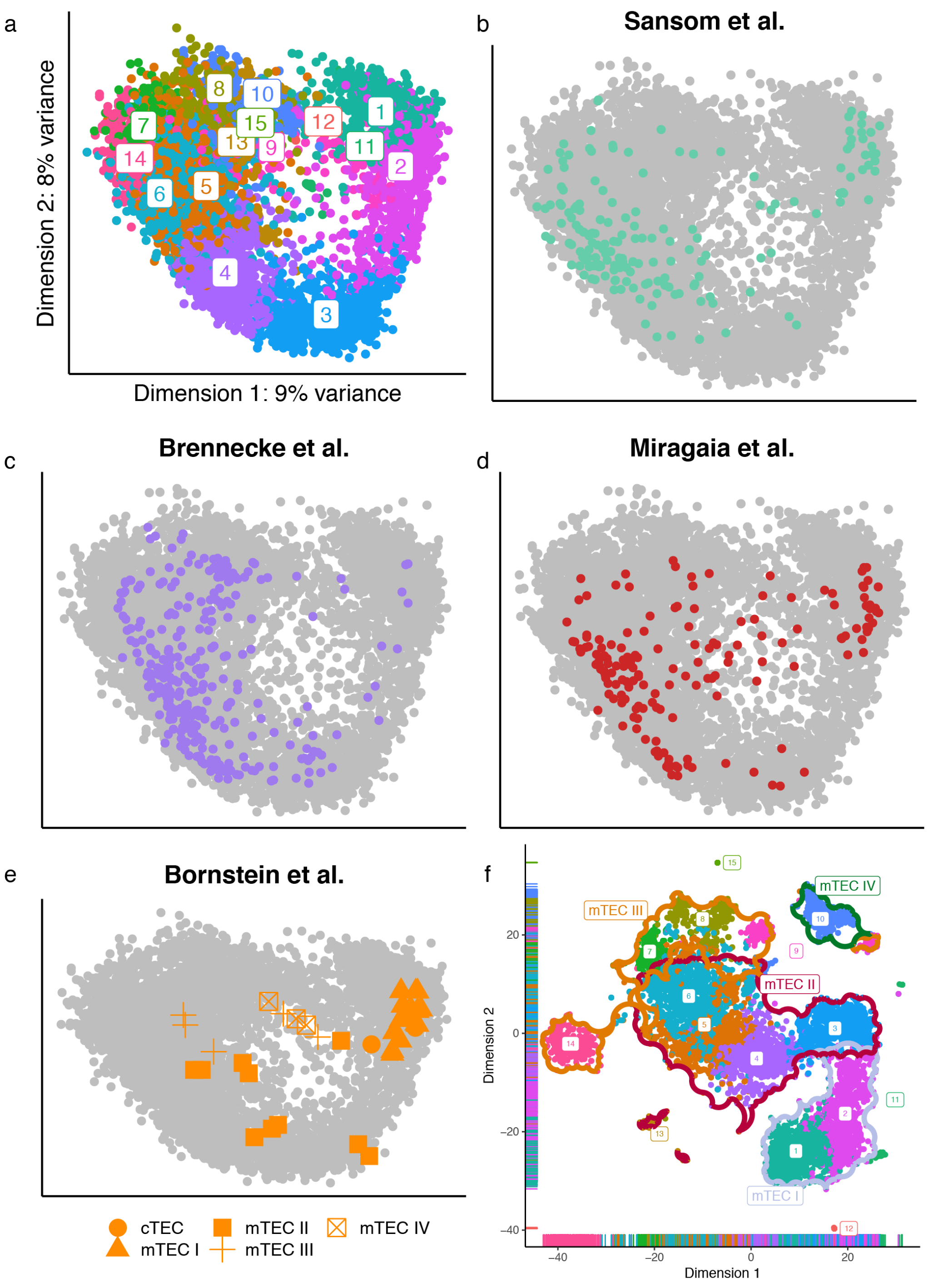

Appendix Figure S3. Cell sub-populations are robustly identified across multiple TEC datasets from the literature.

Visualisations of individual datasets in the literature projected on top of our current combined dataset. (a) PCA visualisation of mTEC sub-populations from the 10x dataset cells are coloured by cluster. (b) PCA visualisation of the Sansom et al. (aquamarine) data projected onto the 10x dataset, (c) Brennecke et al. (purple) dataset projected onto current dataset, and (d) Miragia et al. (brick) dataset projected onto current dataset, (e) Bornstein et al (orange) dataset projected onto current experiment. (b-e) Each dot is a cell coloured by experiment with the grey dots representing the cells from the 10x experiment. (f) tSNE visualisation of the 10x dataset with corresponding mTECI-IV classes from Bornstein et al overlaid as outlines.

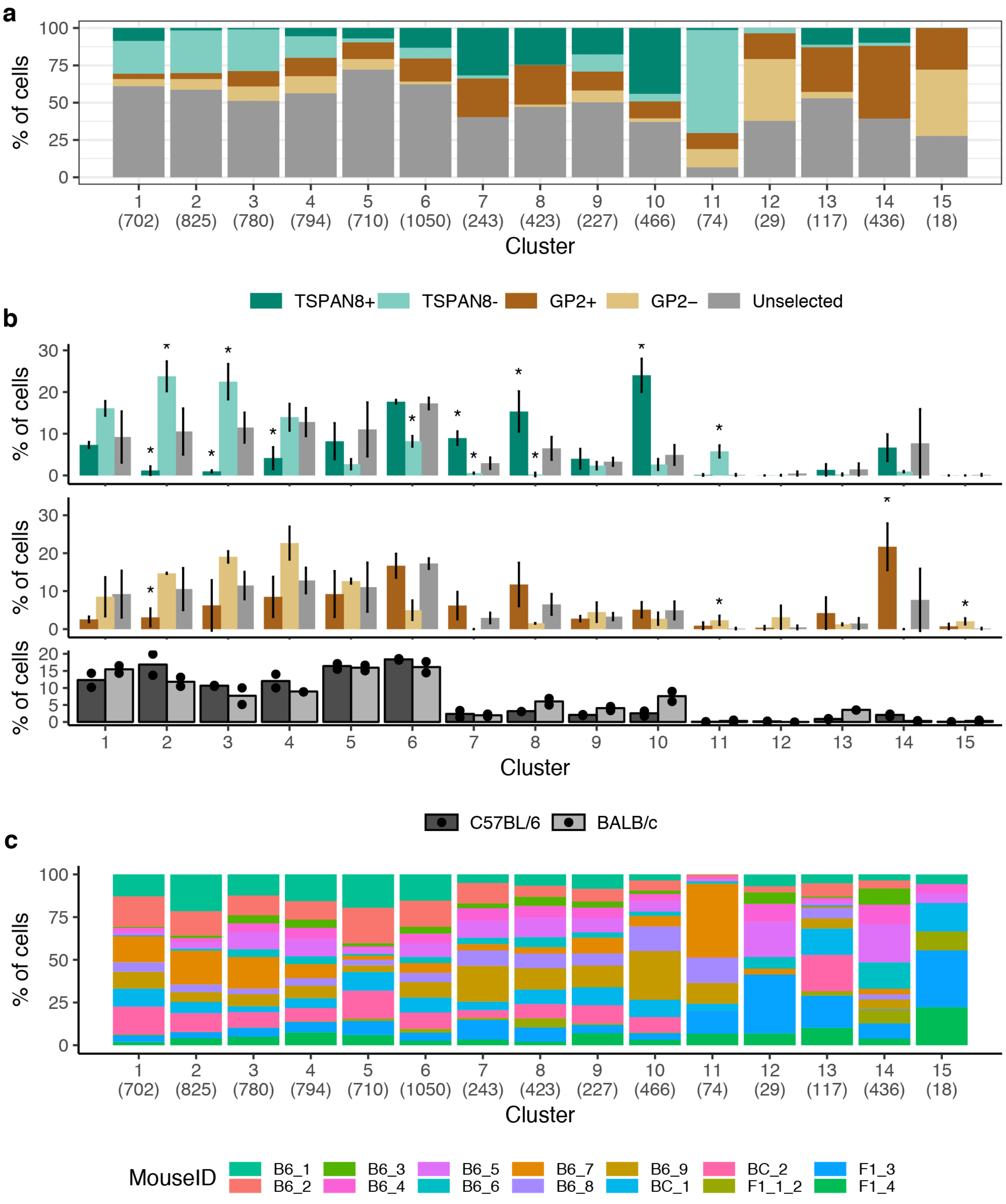

Appendix Figure S4. Contribution to mTEC clusters.
(a) Count of cells within the defined clusters is shown in parenthesis. The FACS condition of cells within each cluster is shown as a stacked bar chart. (b) Bar plot showing the % of cells from each sample that fall within each cluster. Bars are coloured by FACS sort condition as listed in (a), clusters that have a significant ($p<0.05$; Wilcoxon test) difference in mean frequency as compared to unselected mTEC are annotated with a *. (top) TSPAN+/- vs unselected mTEC (middle) GP2+/- vs unselected mTEC (bottom) Comparison of C57BL/6 vs BALB/c unselected mTEC. (c) Count of cells from different mice within each cluster shows that multiple mice contribute to all clusters.

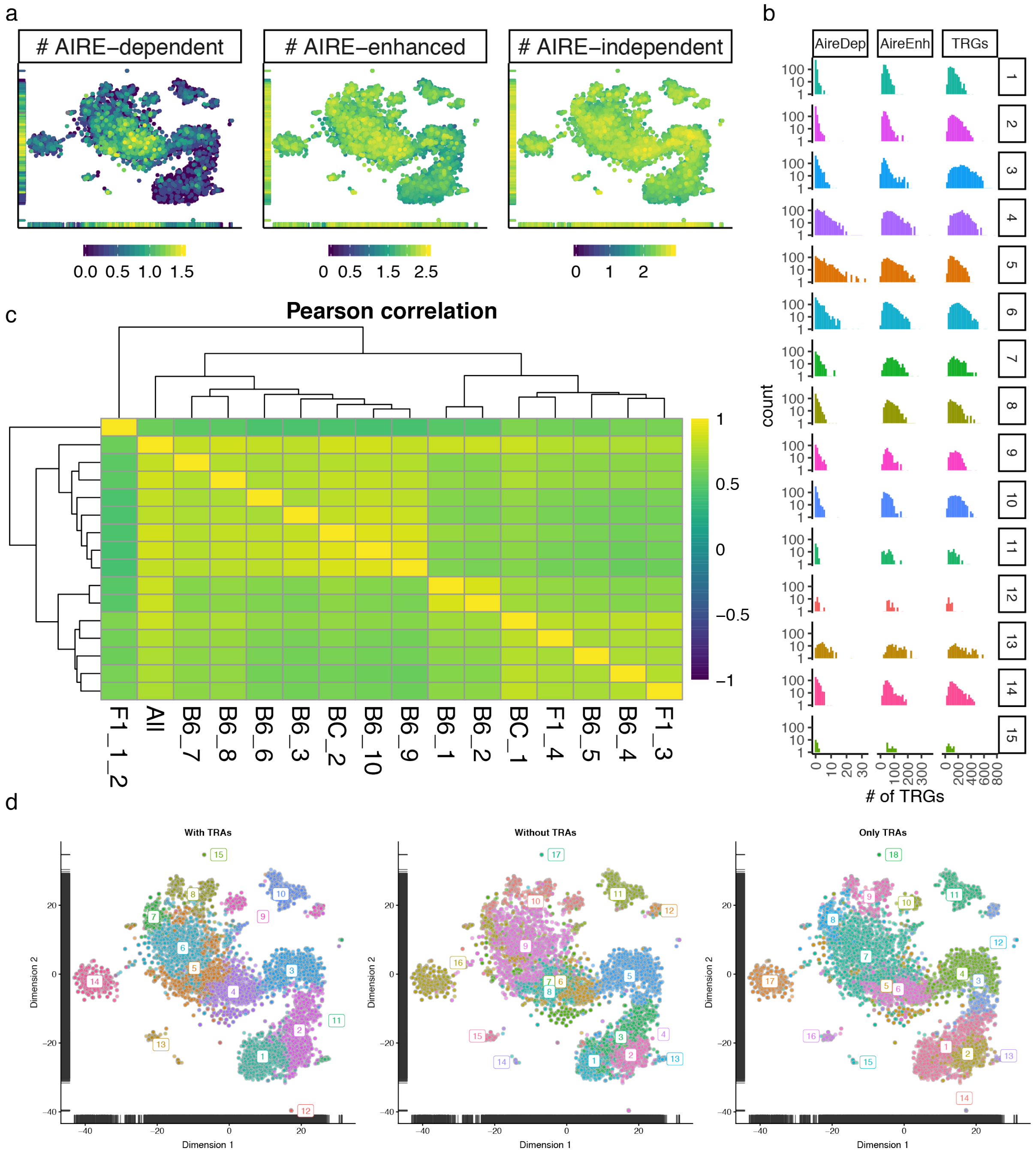

Appendix Figure S5. Contribution to mTEC clusters.
(a) Log10 of the number (#) of AIRE-dependent, AIRE-enhanced and AIRE-independent TRGs expressed per cell visualised on a tSNE plot. (b) Histogram showing the number (#) of AIRE-dependent, AIRE-enhanced and AIRE-independent TRGs expressed per cell within each cluster. (c) Pearson correlation of co-expression frequency for pairs of TRGs (AIRE-regulated and AIRE-independent) between individual mice or all mice pooled together (see Table S1 for mouse identifiers) from mature mTEC clusters only (clusters 3-6) (d) Clusters identified using all genes (left), without any TRAs (middle), or with only TRAs (right). These alternative analyses produced highly similar clusterings of the 6,894 mTEC (AMI 0.63: no TRGs and AMI 0.68: only TRGs).

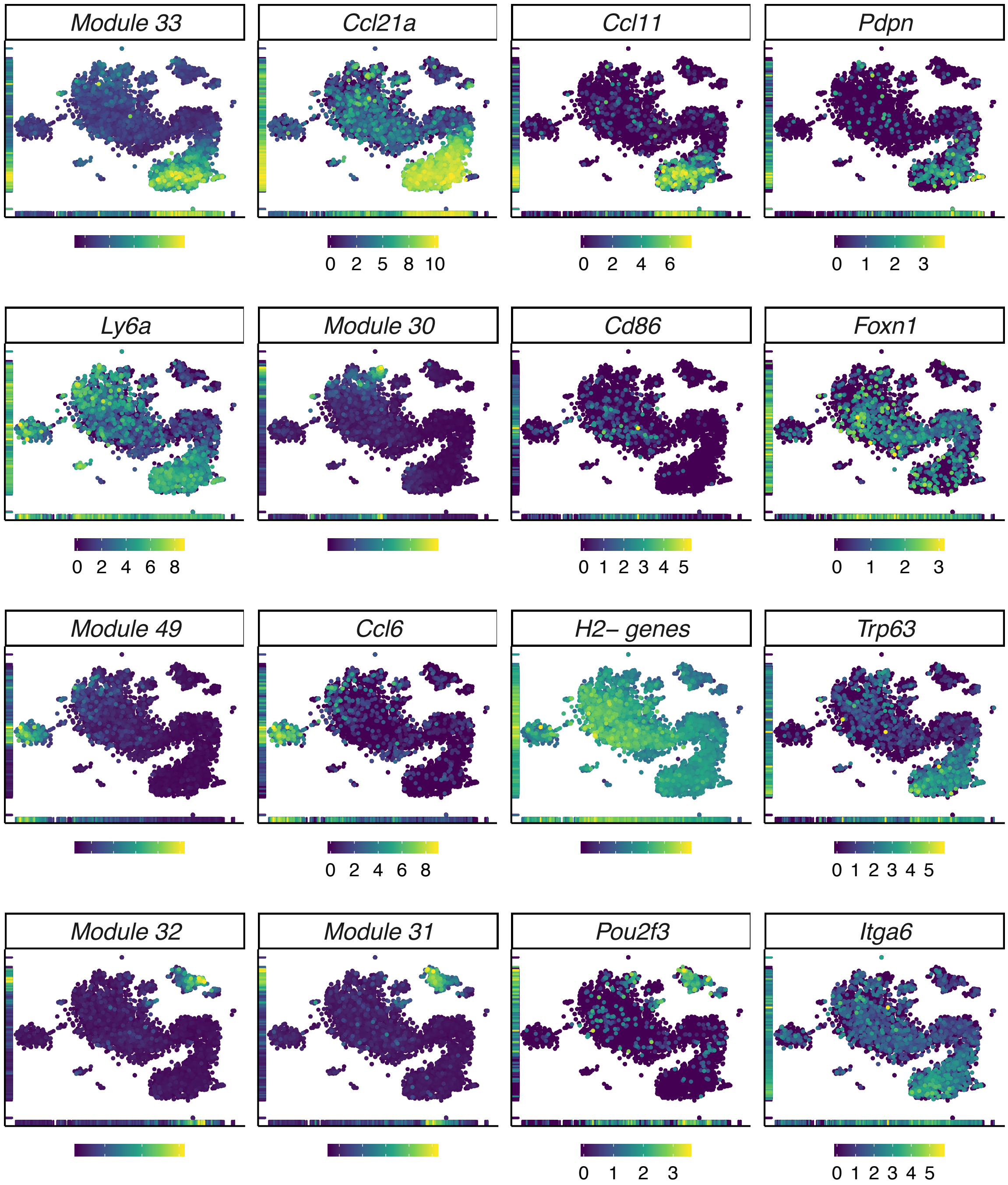

Appendix Figure S6. Expression of selected genes across the dataset.
t-SNE visualisations of Log2 expression of selected genes or aggregated expression of genes within a selected module. Colour bar and scale below each plot indicate the level of expression of the gene or module within those cells.

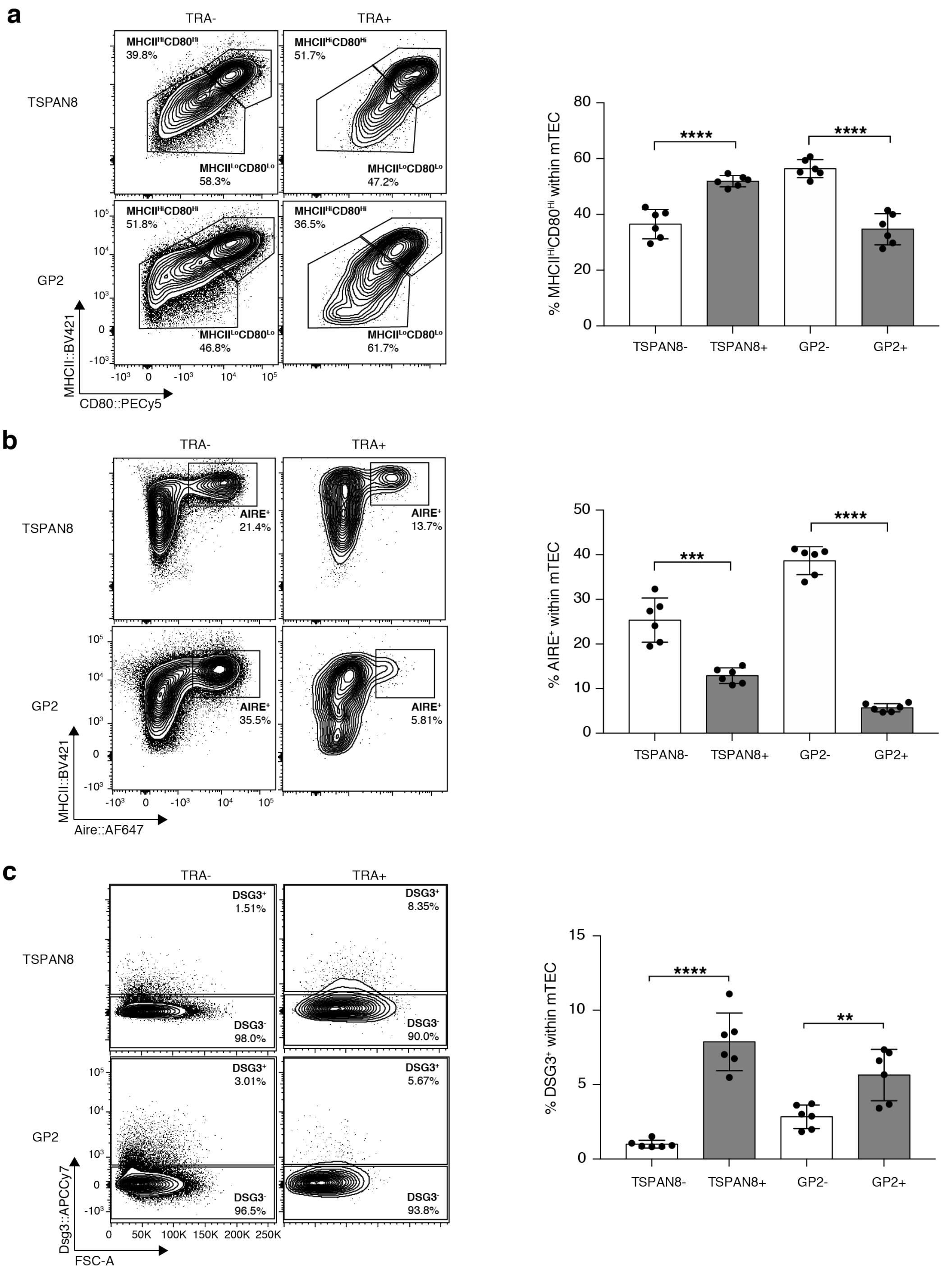

Appendix Figure S7. mTEC expressing the enhanced TRAs Gp2 and Tspan8 have features of terminal differentiation.

mTEC ectopically expressing Gp2 and Tspan8 on their cell surface show features of terminal differentiation compared to their TRA- counterparts such as decreased expression of (**a)** MHCII and CD80 (Gp2+ mTEC only) and (**b)** Aire, and (**c)** increased expression of Dsg3 (gates set on basis of an isotope control antibody). Data are shown as bar graphs showing mean frequency (+/- sd) alongside representative FACS plots; n=6, across 2 independent experiments.

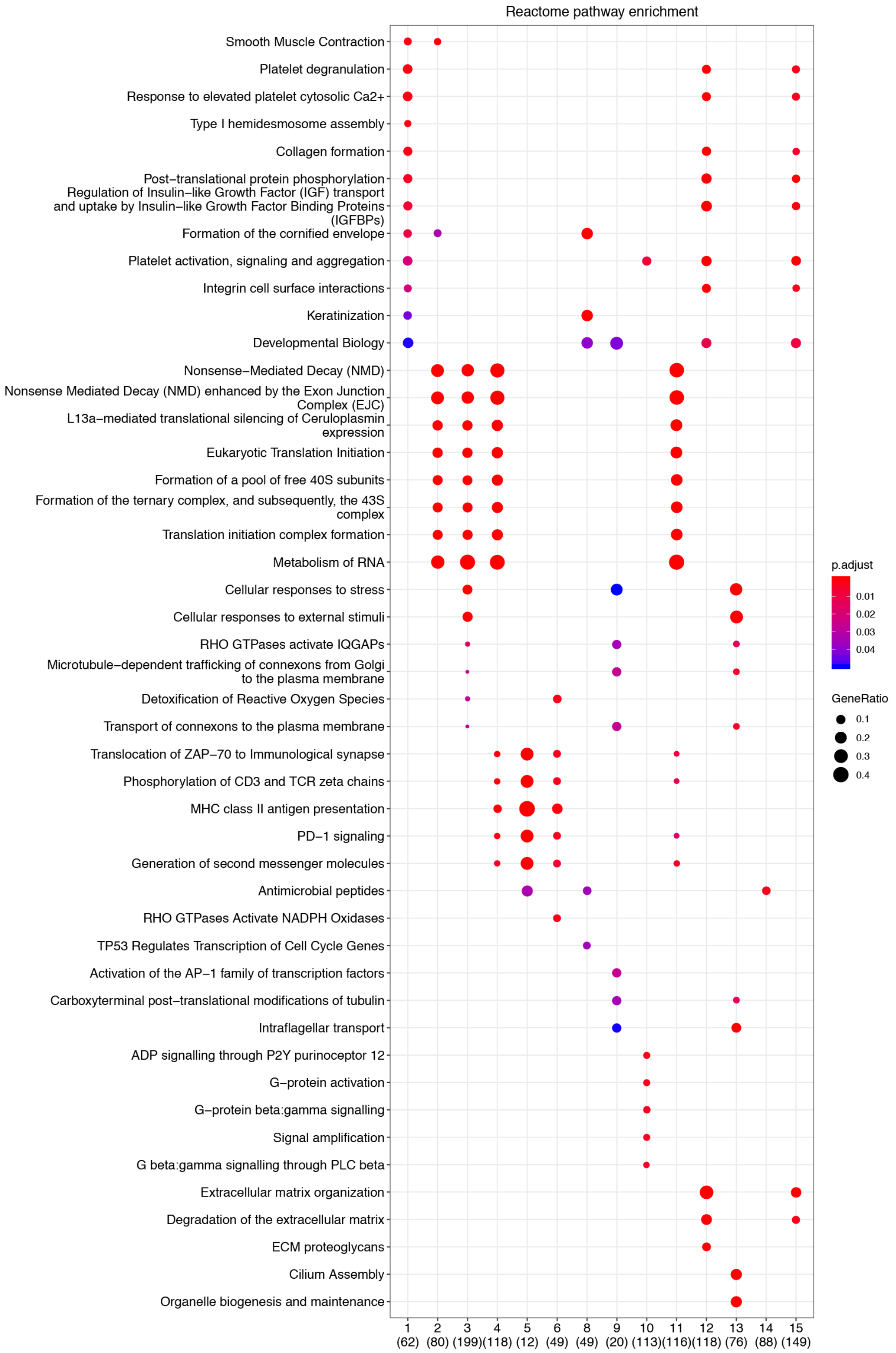

Appendix Figure S8. Enriched pathways in mTEC clusters.

Adjusted p-value of enrichment for marker genes from each cluster in the Reactome pathway database. Gene ratio is the fraction of pathway genes in query set.

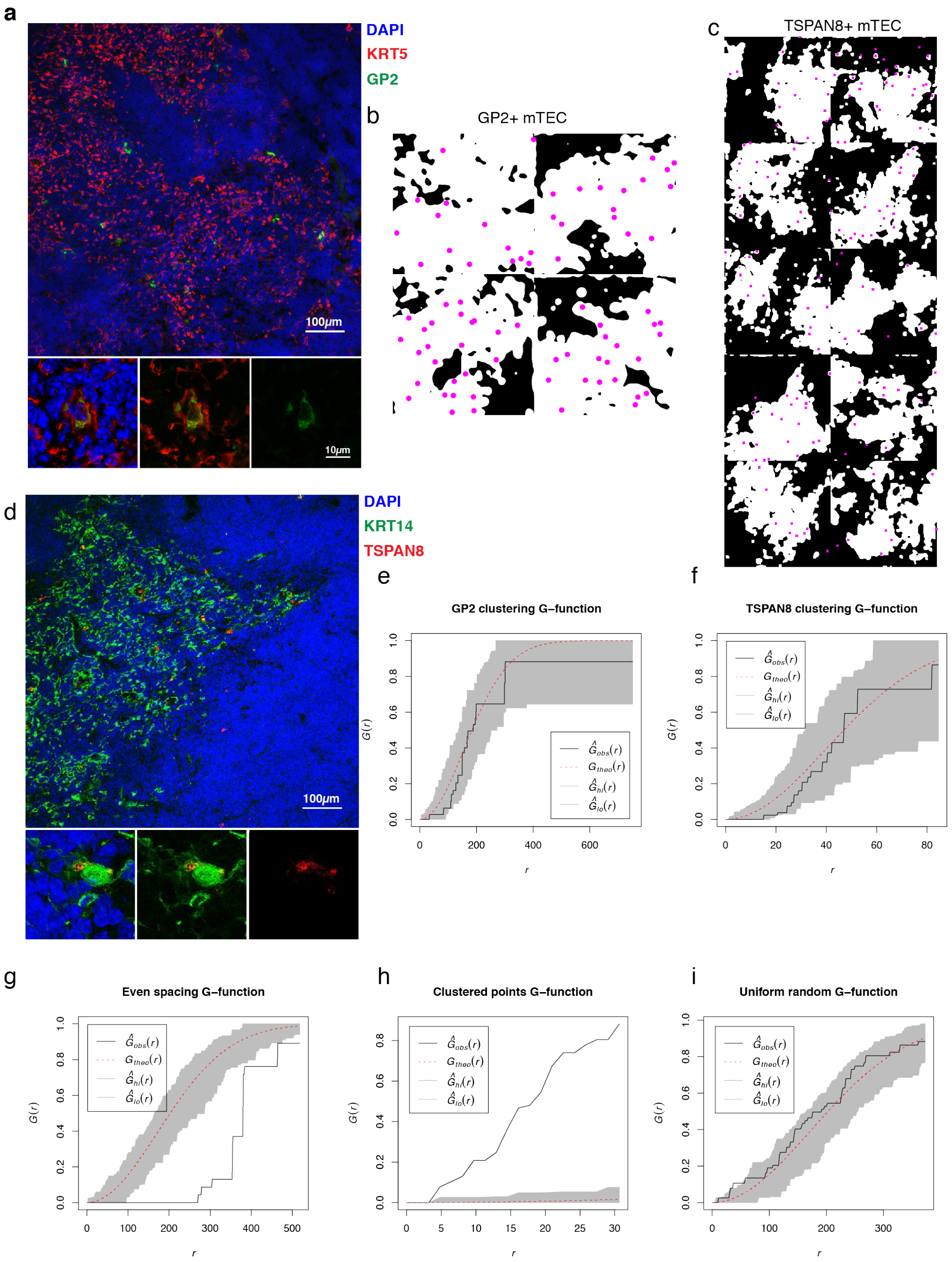

Appendix Figure S9: Spatial analysis of Gp2 distribution shows that Gp2+ mTEC are randomly distributed within the thymic medulla.

(a) Representative microscope image of a thymic slice. GP2 is stained in green and KRT5 (mTEC) in red. Zoomed images show the overlap of GP2 and KRT5 for one GP2+ mTEC (repeat of Figure 8a). (b) Image mask showing KRT5+ regions of 4 thymic slices (in white) with magenta dots at the location of GP2+ mTEC (similar to figure 8c: the mask in the lower-left corresponds to the image in panel a but should be inverted). (d) Image mask showing KRT14+ regions of 10 thymic slices (in white) with magenta dots at the location of TSPAN8+ mTEC (repeat of Figure 8c: TSPAN8+ masks: note the 4th image on the left corresponds to the image in panel d). (d) Representative microscope image of a thymic slice. TSPAN8 is stained in red and KRT14 (mTEC) in green. Zoomed images show the overlap of TSPAN8 and KRT14 for one TSPAN8+ mTEC. (e-i) G-function observed vs expected for GP2 spacing (e), TSPAN8 spacing (f) or simulated spacings (g-i). G-function: cumulative distribution of the distance between a random point and its nearest neighbour. Deviation of the Gobs(r) from Gtheo(r) suggests that the points or closer or farther than expected of a random process.
